# Supplementary figures and images for: Plant host and drought shape the root associated fungal microbiota in rice
Source: PeerJ. 2019 Sep 11;7:e7463. doi: 10.7717/peerj.7463 (PMC6744933; doi:10.7717/peerj.7463)

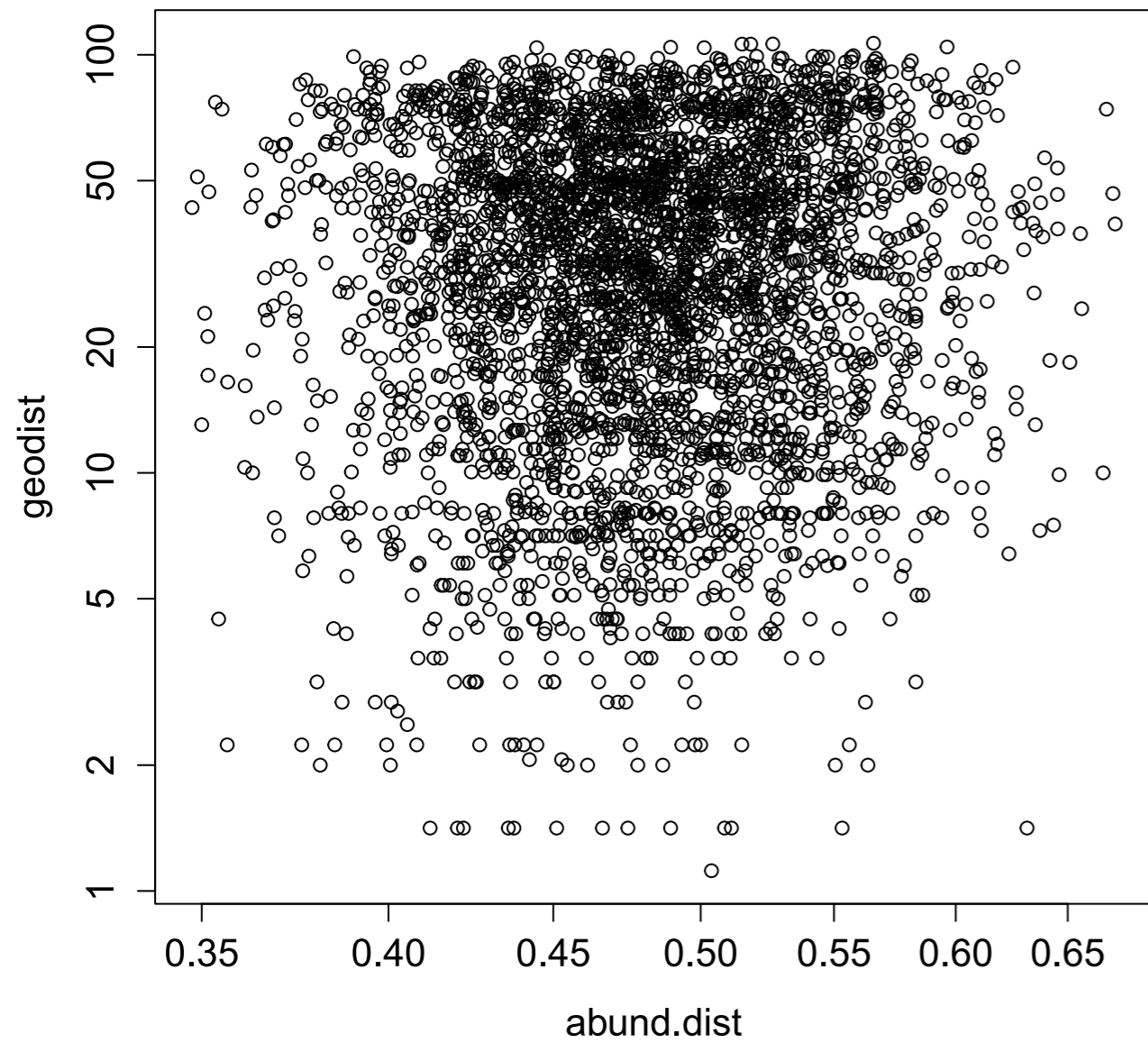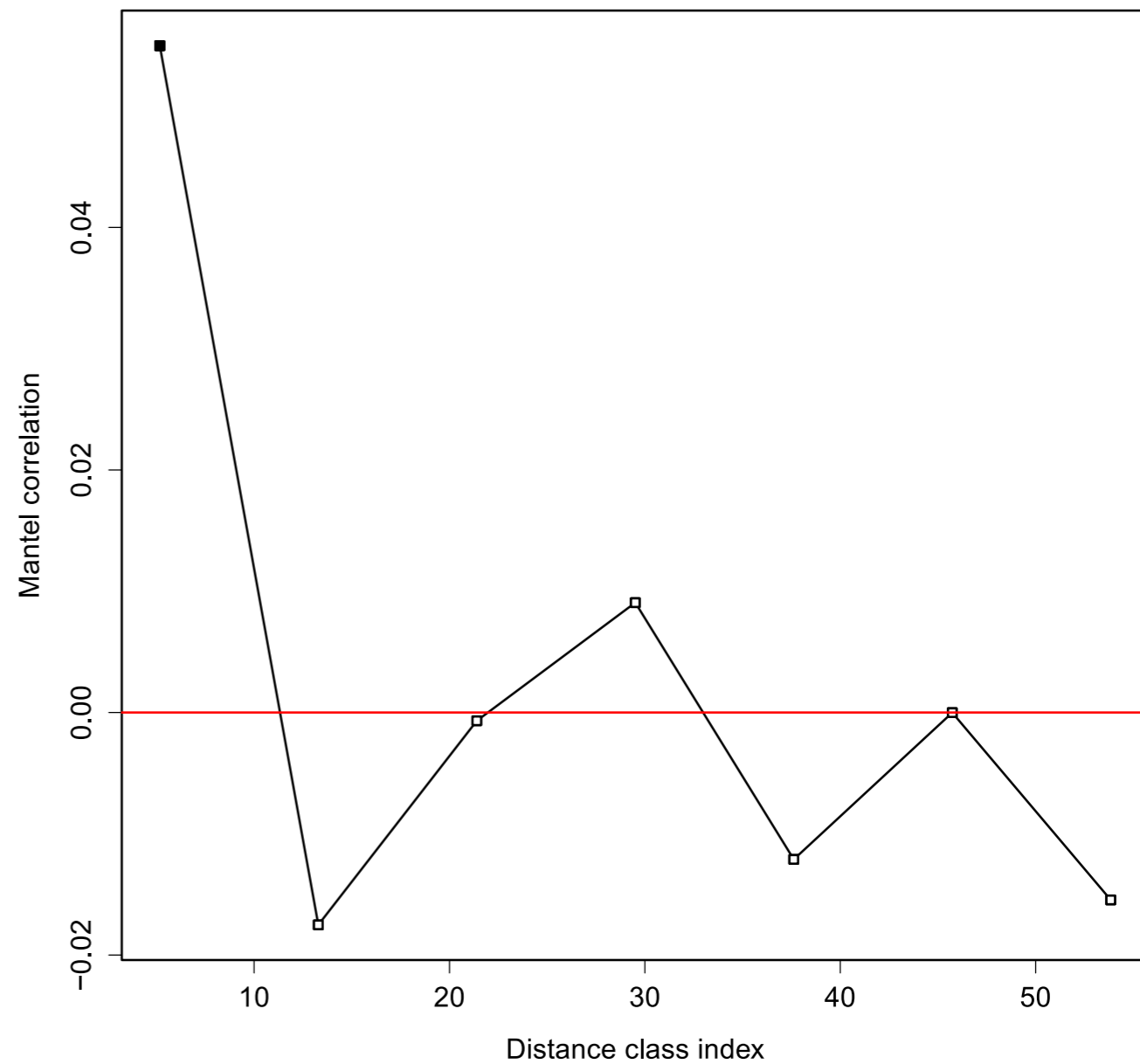

Supplement: Figure S1 — To study the field position effect on the fungal community results. Euclidean distances between samples (left) and correlogram (right) for all samples. There is no correlation between field position and fungal community, hence no field effect was found. [file peerj-07-7463-s001.pdf]

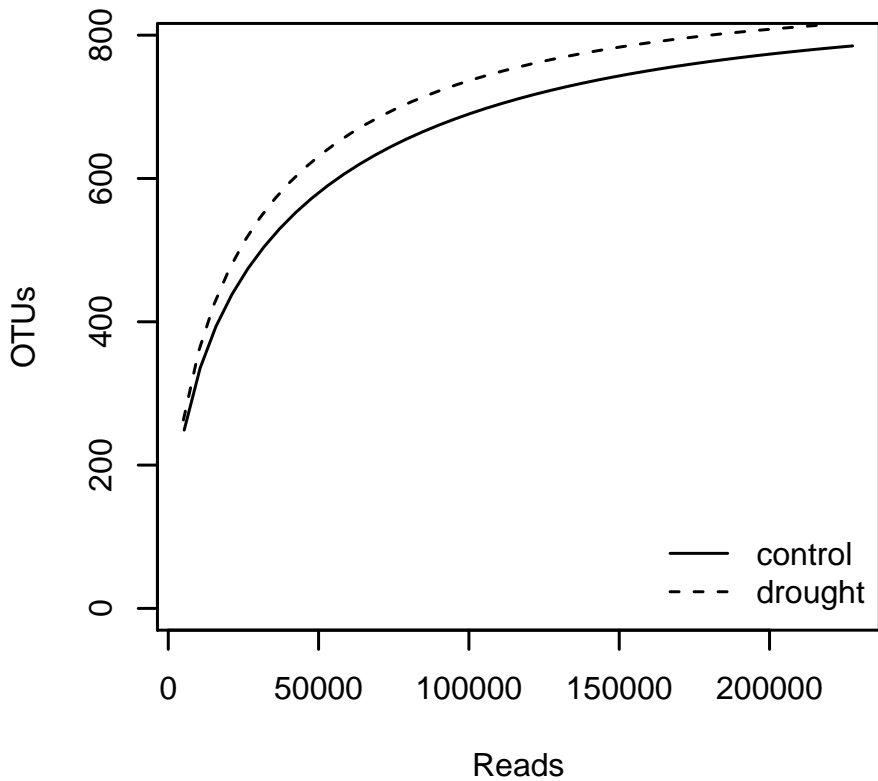

Supplement: Figure S2 — It represents the number of fungal OTUs found for the two treatments, control and drought. [file peerj-07-7463-s002.pdf]

***Drought***

***77***

***737***

***48***

***Control***

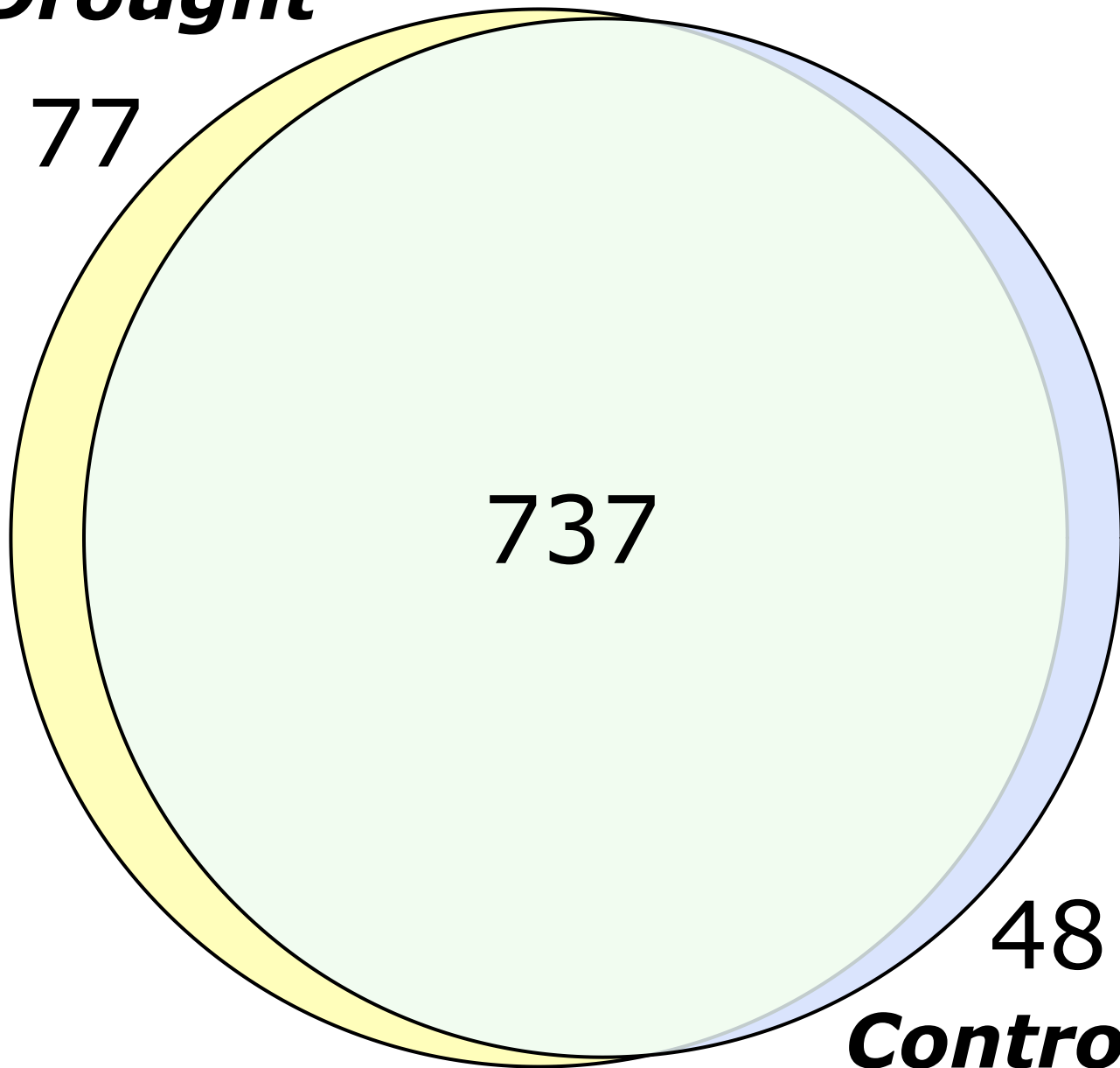

Supplement: Figure S4 — Number of OTUs found in control treatment only, in drought treatment only and in both (i.e., γ-diversity). The 40 rarest OTUs were removed from this analysis. [file peerj-07-7463-s004.pdf]

(a)

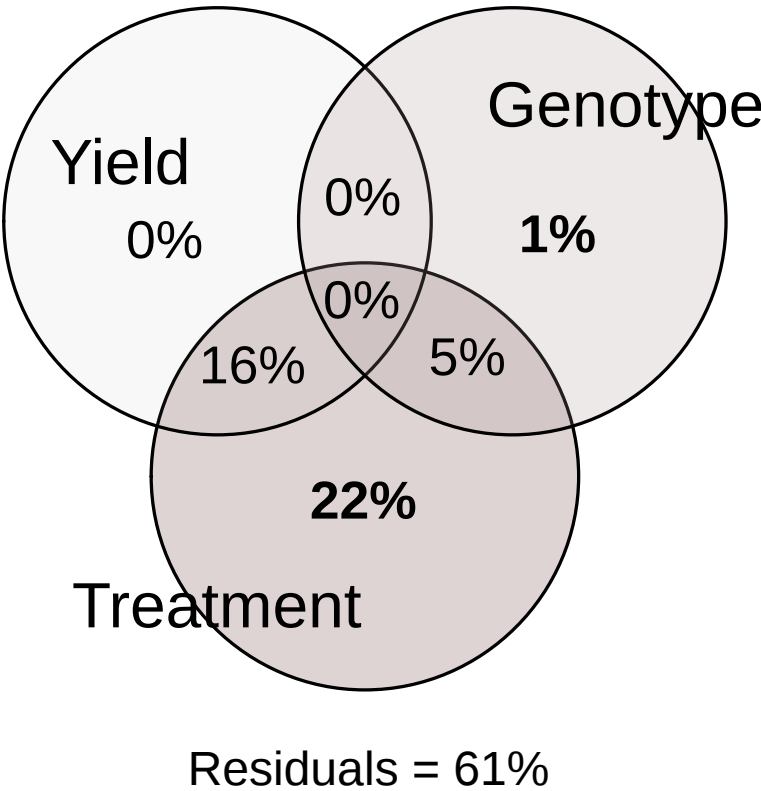

(b)

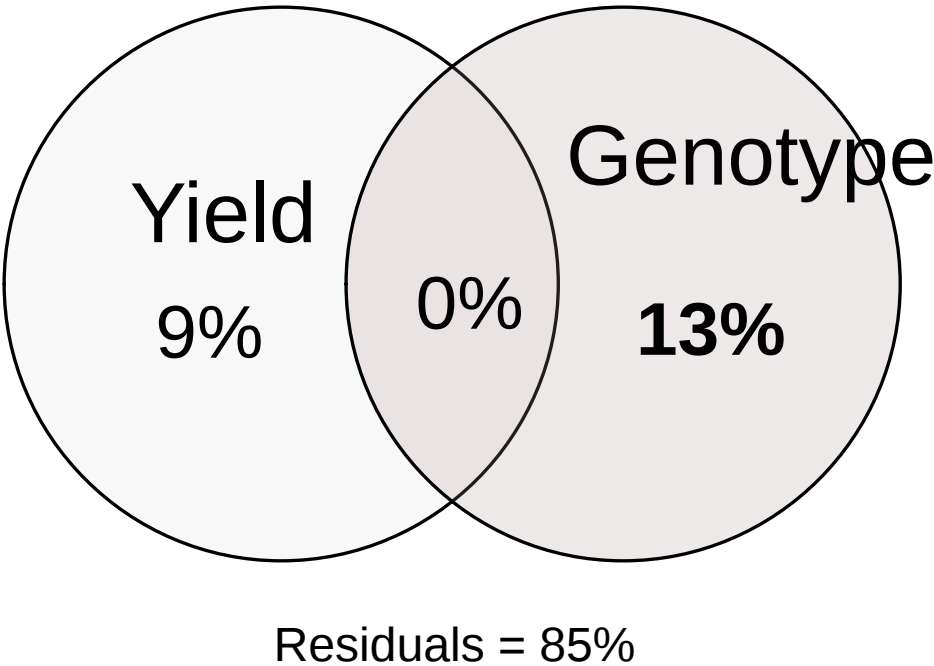

Supplement: Figure S5 — It is illustrated using Venn diagrams. Each partition represents the variation due to one environmental factor affecting the fungal community abundance. In bold are the adjusted R2 values for independent factors that contribute significantly. (A) Treatment explains 22% of the variation in community structure (P = 0.001). Combined, yield and treatment can explain 16% of the variation in community structure (P = 0.001). (B) VPA with the ‘robustness’ data for yield and the OTUs abundance under drought shows that 13% of the community variation is due to a genotype effect (P = 0.002). Data with yield ‘robustness’ and OTU abundance under control shows a significant 5% of explanation by the genotype (P = 0.05) but not by the yield (not shown). [file peerj-07-7463-s005.pdf]

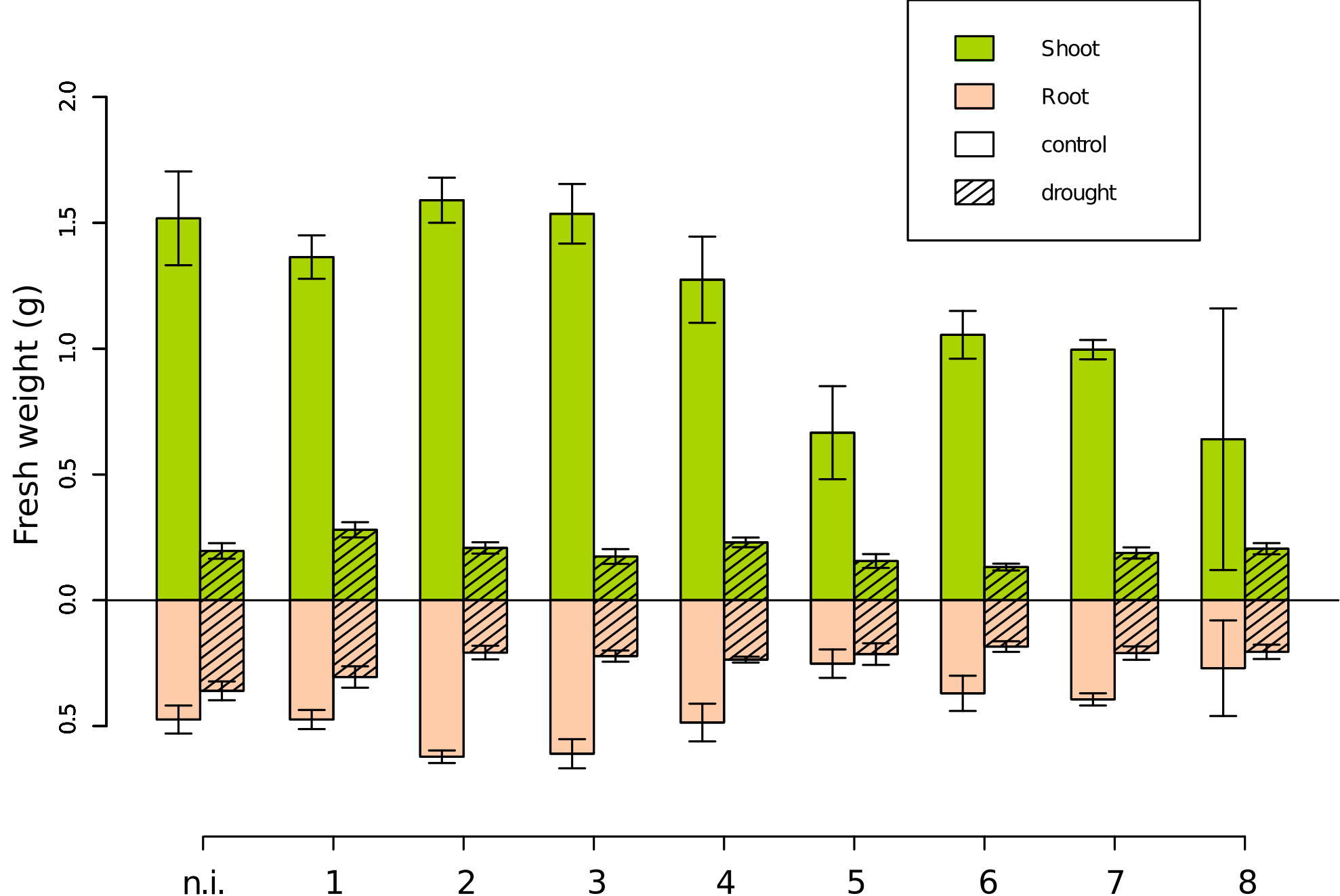

Supplement: Figure S6 — There is no effect of inoculation on plant shoot biomass with any of the fungal strains under control nor drought conditions. [file peerj-07-7463-s006.pdf]

**control**

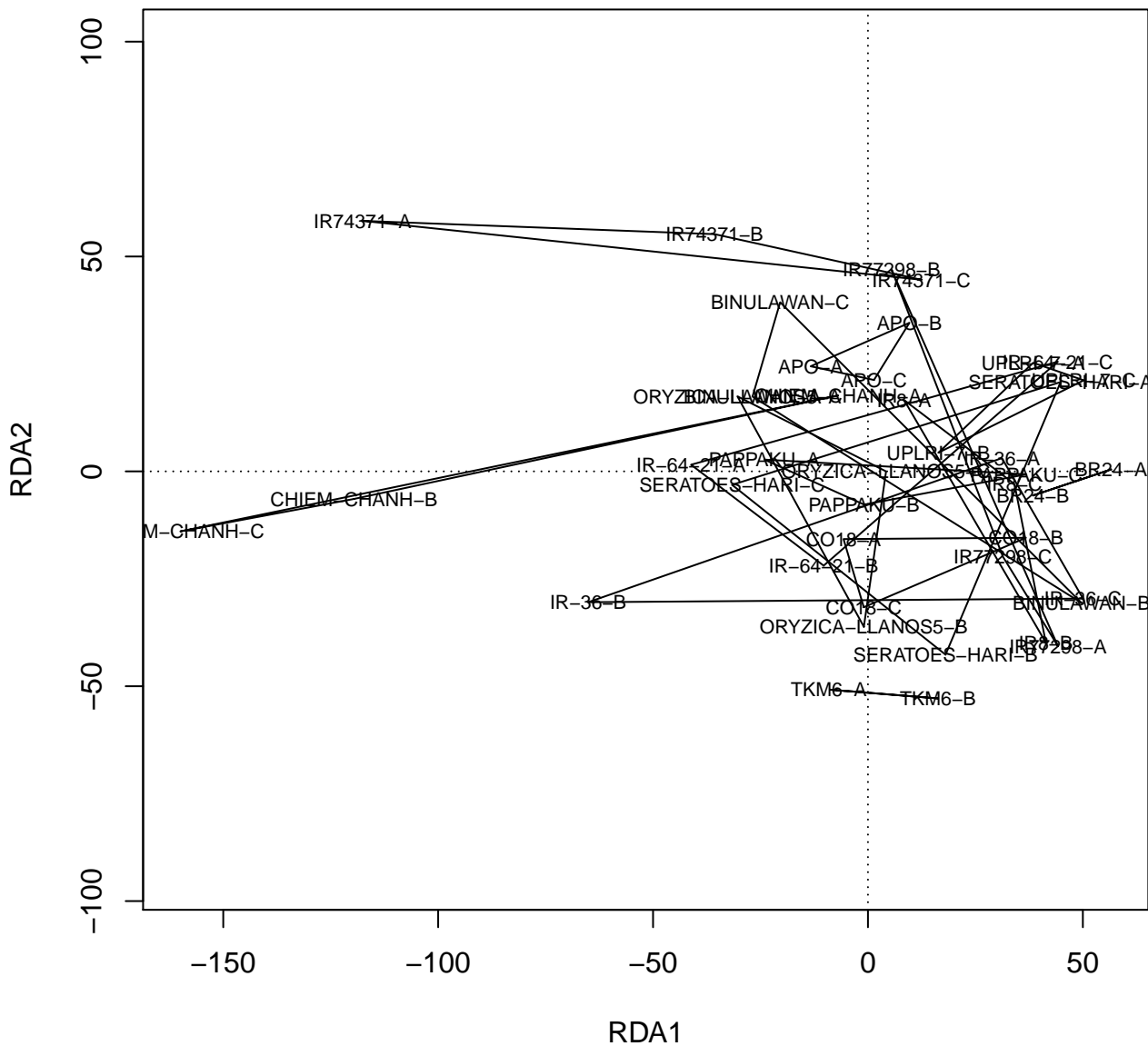

# drought

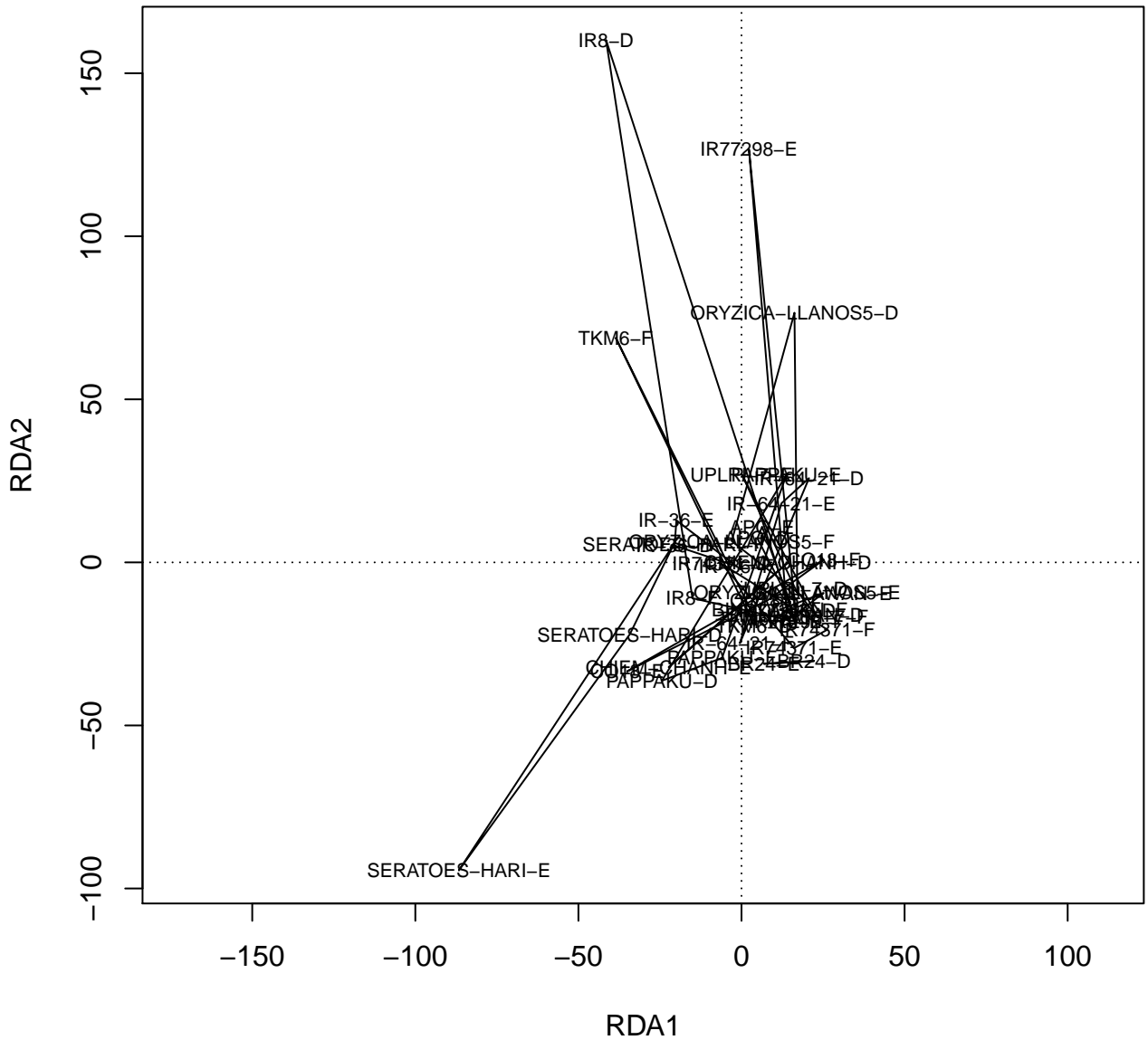

# Control (robustness data)

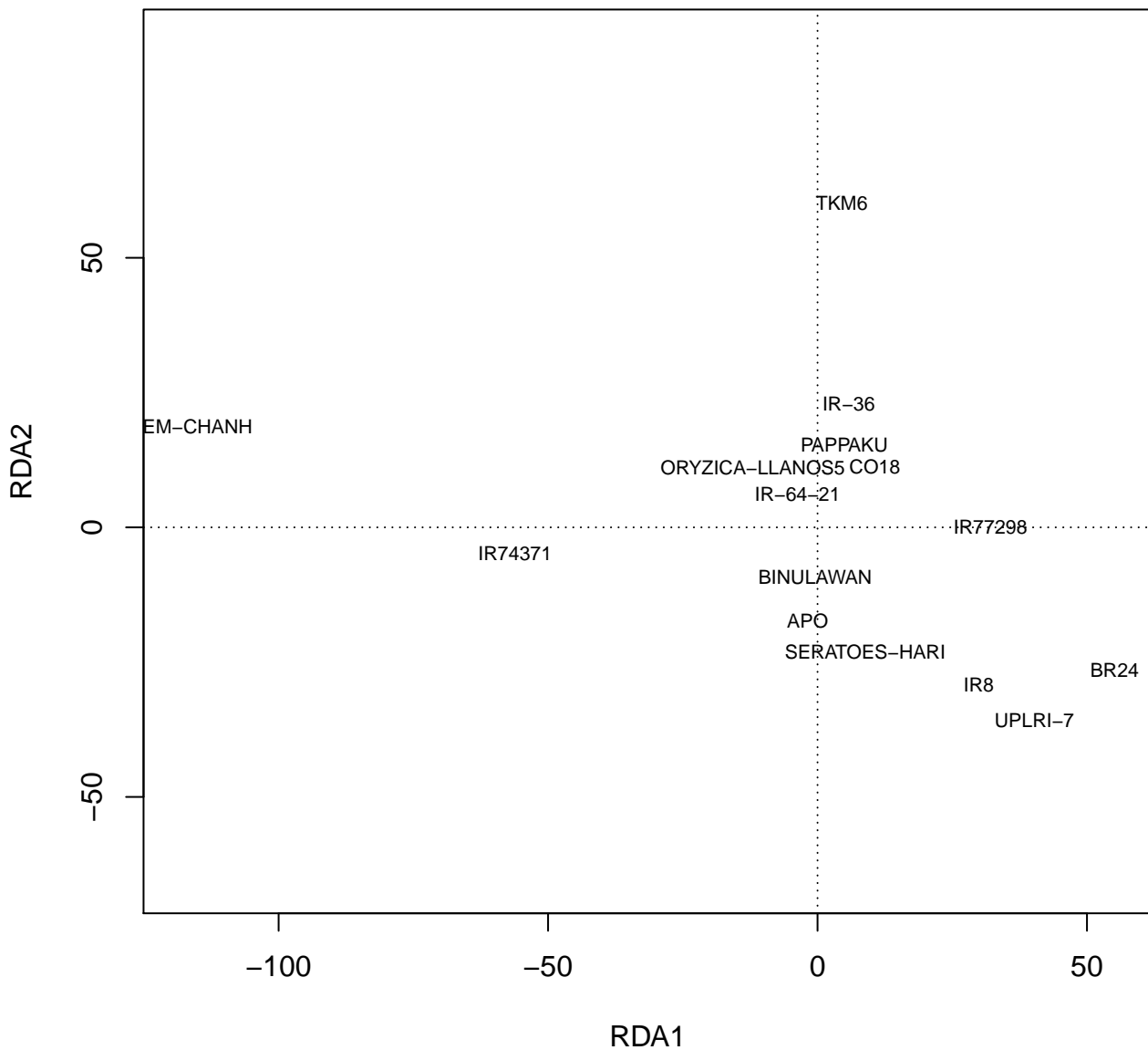

# Drought (robustness data)

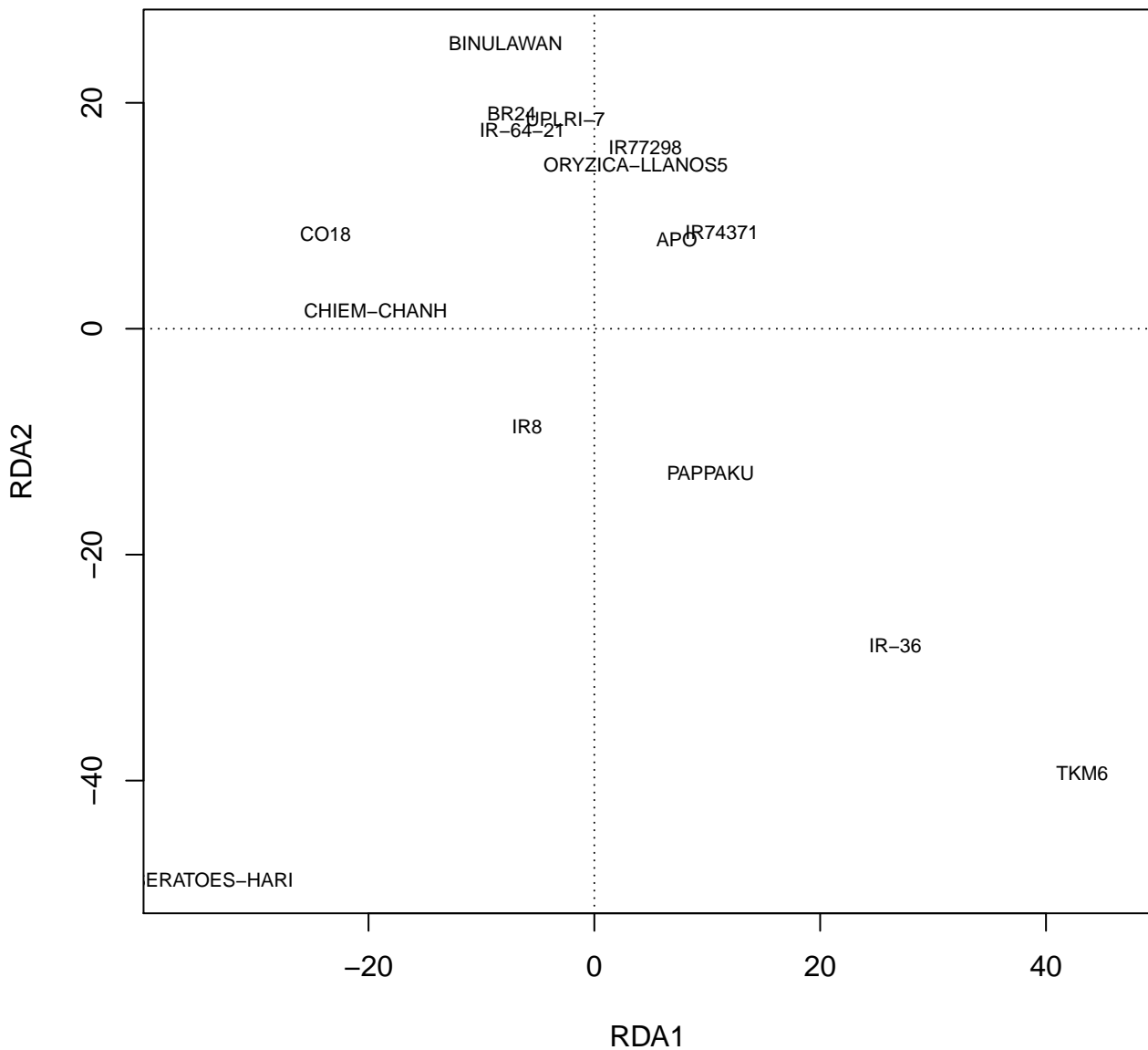

Supplement: Supplemental Information 2 [file peerj-07-7463-s012.pdf]
