# Supplementary material for: Plant host and drought shape the root associated fungal microbiota in rice
Source: PeerJ. 2019 Sep 11;7:e7463. doi: 10.7717/peerj.7463 (PMC6744933; doi:10.7717/peerj.7463)
Supplement: Figure S3 — Ascomycota (top) and Basidiomycota (bottom). For each tree the different classes are represented. [file peerj-07-7463-s003.pdf]

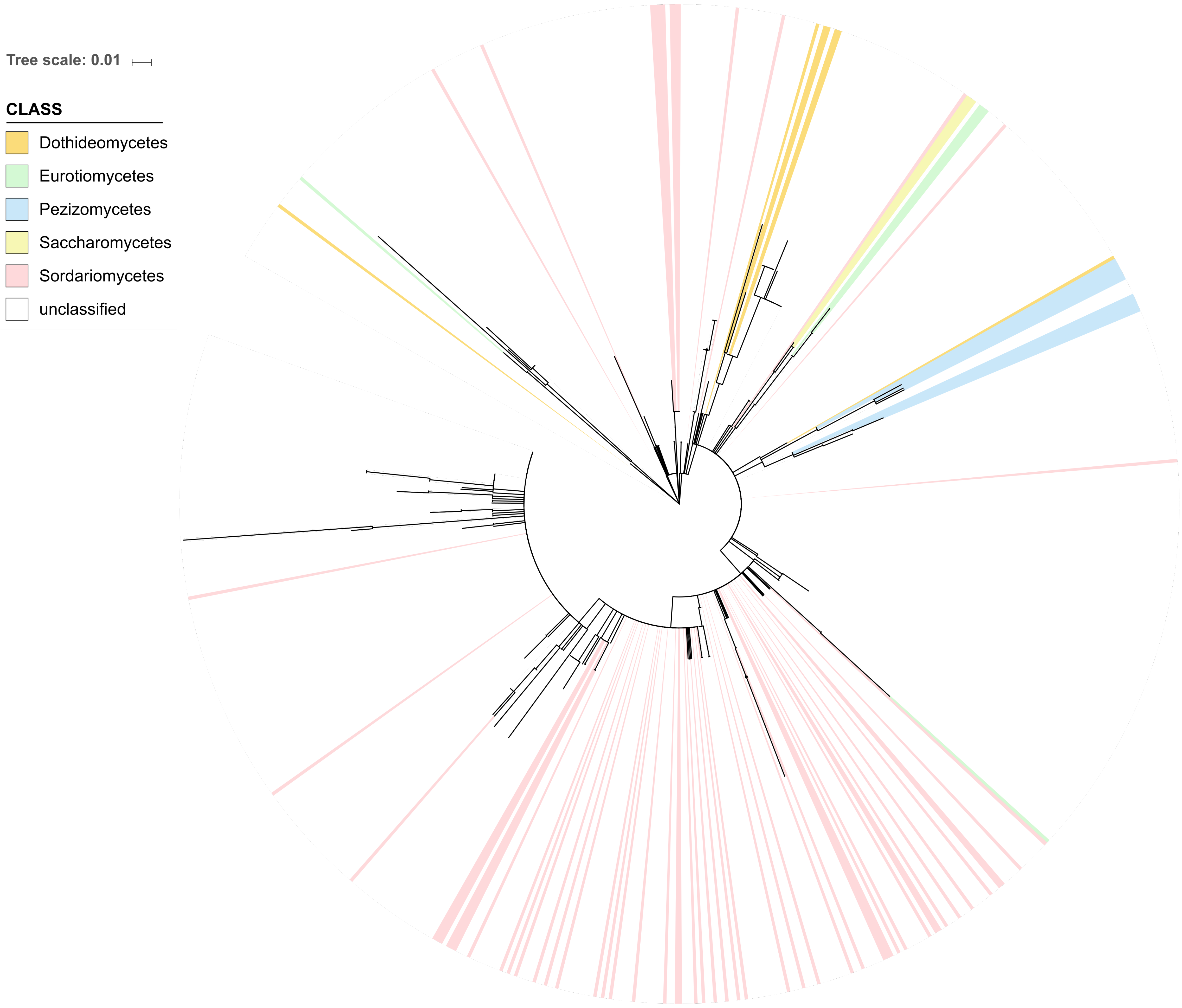
Tree scale: 0.01
CLASS
Dothideomycetes
Eurotiomycetes
Pezizomycetes
Saccharomycetes
Sordariomycetes
unclassified

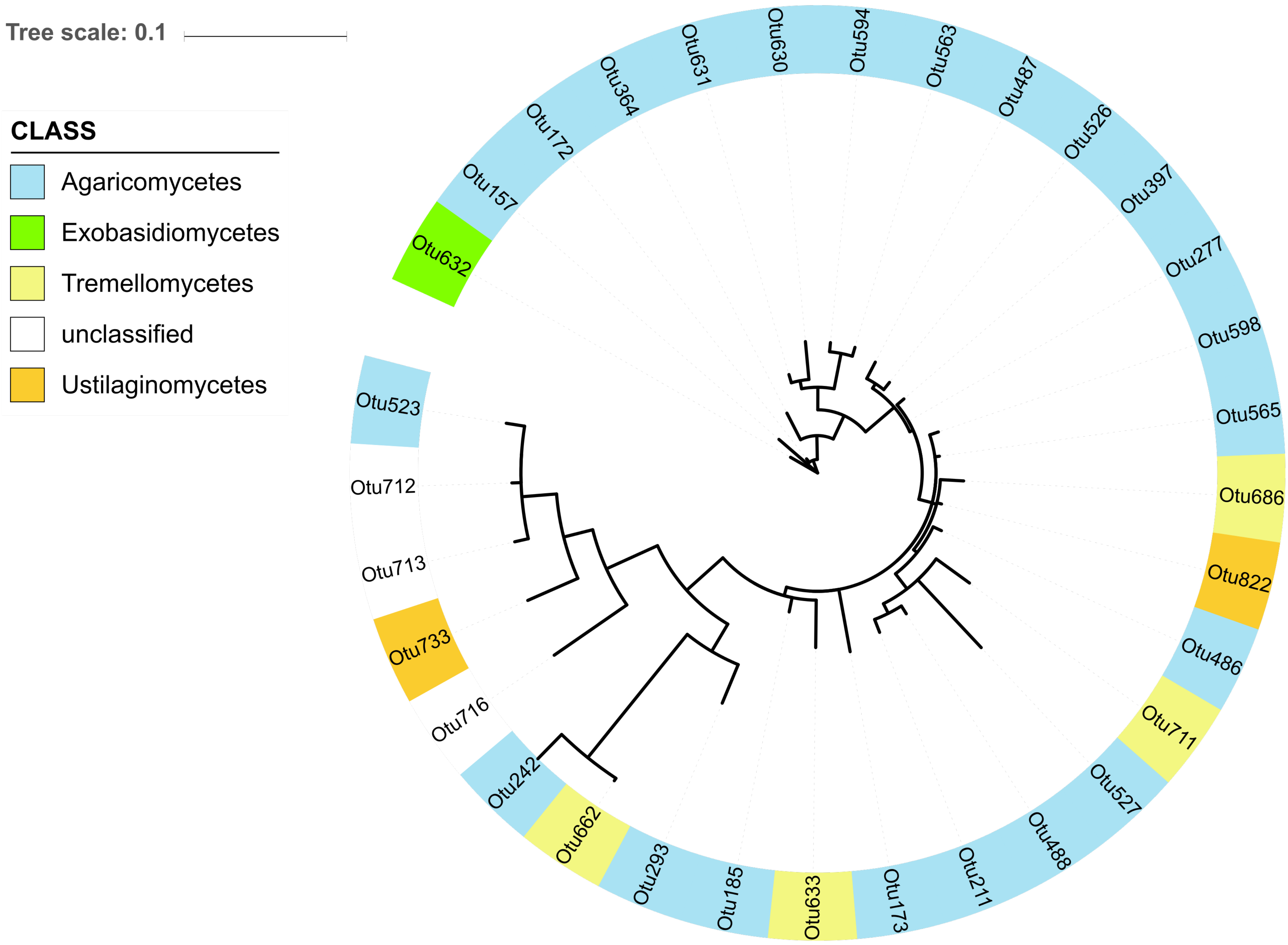
Tree scale: 0.1
CLASS
Agaricomycetes
Exobasidiomycetes
Tremellomycetes
unclassified
Ustilaginomycetes
Otu630
Otu594
Otu563
Otu487
Otu526
Otu397
Otu277
Otu598
Otu565
Otu686
Otu822
Otu486
Otu711
Otu527
Otu488
Otu211
Otu173
Otu633
Otu185
Otu293
Otu662
Otu242
Otu716
Otu733
Otu713
Otu712
Otu523
Otu632
Otu157
Otu172
Otu364
Otu631
