# Supplementary material for: Plant host and drought shape the root associated fungal microbiota in rice
Source: PeerJ. 2019 Sep 11;7:e7463. doi: 10.7717/peerj.7463 (PMC6744933; doi:10.7717/peerj.7463)
Supplement: Table S1 — Summary of the statistics for the linear model fitted for the interaction between treatment ‘fungus’ (A. phaeospermum inoculated vs. non-inoculated) and ‘treatment’ (control vs. drought) for a number of different plant traits. [file peerj-07-7463-s007.pdf]

Table S1. Summary of the statistics for the linear model fitted for the interaction between treatment 'fungus' (*A. phaeospermum* inoculated vs. non-inoculated) and 'treatment' (control vs. drought) for a number of different plant traits.

Shoot FW	DF	Sum sq	Mean sq	***F*-value**	***P*-value**
Fungus	9	3.3087	0.3676	7.6568	**<.0001**
Treatment	1	23.4098	23.4098	487.5605	**<.0001**
Fungus:treatment	9	2.1427	0.2381	4.9585	**<.0001**
Residuals	73	3.5050	0.0480		
Shoot DW	DF	Sum sq	Mean sq	*F*-value	*P*-value
Fungus	9	0.1535	0.0170	4.5058	**<.0001**
Treatment	1	0.8526	0.8526	225.1402	**<.0001**
Fungus:treatment	9	0.0924	0.0102	2.7121	**0.008**
Residuals	73	0.2764	0.0037		
Root FW	DF	Sum sq	Mean sq	*F*-value	*P*-value
Fungus	9	0.5866	0.0651	6.3817	**<.0001**
Treatment	1	1.1844	1.1844	115.972	**<.0001**
Fungus:treatment	9	0.3509	0.0389	3.8177	**0.0005**
Residuals	73	0.7455	0.0102		
Root DW	DF	Sum sq	Mean sq	*F*-value	*P*-value
Fungus	9	0.0654	0.0072	3.7445	**0.0006**
Treatment	1	0.0738	0.0738	37.9897	**<.0001**
Fungus:treatment	9	0.023	0.0025	1.3264	0.2384
Residuals	73	0.1418	0.0019		
Shoot Water	DF	Sum sq	Mean sq	*F*-value	*P*-value
Fungus	9	2.0961	0.2329	8.2902	**<.0001**
Treatment	1	15.3271	15.3271	545.5900	**<.0001**
Fungus:treatment	9	1.3612	0.1512	5.3836	**<.0001**
Residuals	73	2.0508	0.0281		
Root : Shoot FW	DF	Sum sq	Mean sq	*F*-value	*P*-value
Fungus	9	2.4403	0.2711	0.9951	0.4518
Treatment	1	21.5029	21.5029	78.9168	**<.0001**
Fungus:treatment	9	2.9660	0.3296	1.2095	0.3025
Residuals	73	19.8907	0.2725		
Root : Shoot DW	DF	Sum sq	Mean sq	*F*-value	*P*-value
Fungus	9	11.4380	1.2710	0.7615	0.6518
Treatment	1	55.9060	55.9060	33.5010	**<.0001**
Fungus:treatment	9	11.9270	1.3250	0.7941	0.6226
Residuals	73	121.8210	1.6690		

Significant *P*-values are indicated in bold. FW: fresh weight; DW: dry weight; DF: degrees of freedom; Sum sq: sum of squares; Mean sq: mean of squares.
