# Supplementary material for: Plant host and drought shape the root associated fungal microbiota in rice
Source: PeerJ. 2019 Sep 11;7:e7463. doi: 10.7717/peerj.7463 (PMC6744933; doi:10.7717/peerj.7463)
Supplement: Table S2 — Summary of the ANOVA statistics in the fitted linear model for the different A. phaeospermum strains effect on fresh root:shoot ratio. [file peerj-07-7463-s008.pdf]

Table S2. Summary of the ANOVA statistics in the fitted linear model for the different *A. phaeospermum* strains effect on fresh root:shoot ratio.

Control	Estimate	Std. error	*t*-value	*P*(>|t|)
(Intercept)	0.3392	0.2334	1.4530	0.1505
Strain 1	0.0177	0.3301	0.0540	0.9573
Strain 2	0.0585	0.3301	0.1770	0.8598
Strain 3	0.0618	0.3301	0.1870	0.8518
Strain 4	0.0453	0.3301	0.1370	0.8912
Strain 5	0.0739	0.3301	0.2240	0.8233
Strain 6	0.0083	0.4367	0.0190	0.9848
Strain 7	0.0570	0.3301	0.1730	0.8632
Strain 8	0.1924	0.4367	0.4410	0.6608
Drought	Estimate	Std. error	*t*-value	*P*(>|t|)
(Intercept)	1.6698	0.3301	5.0580	**<.0001**
Strain 1	–0.3612	0.4668	–0.7740	0.4415
Strain 2	–1.0452	0.4668	–2.2390	**0.0282**
Strain 3	–0.7106	0.4668	–1.5220	0.1323
Strain 4	–1.0125	0.4668	–2.1690	**0.0334**
Strain 5	–0.3775	0.4668	–0.8090	0.4214
Strain 6	–0.5702	0.5474	–1.0420	0.3011
Strain 7	–0.9423	0.4668	–2.0180	**0.0472**
Strain 8	–1.2077	0.5597	–2.1580	**0.0343**

Data under control and drought treatments are shown. Significant *P*-values are indicated in bold text. Std. error: standard error.
