# Supplementary material for: Plant host and drought shape the root associated fungal microbiota in rice
Source: PeerJ. 2019 Sep 11;7:e7463. doi: 10.7717/peerj.7463 (PMC6744933; doi:10.7717/peerj.7463)
Supplement: Table S3 [file peerj-07-7463-s009.pdf]

Table S3. List of rice cultivars used in this study.

IRGC Nº	Cultivar name	Cultivar Nº	Origin	Latitude	Longitude	Kinship sum
IRGC121705	APO::C1	Cultivar1	Philippines	na	na	8.538055648
IRGC117659	BINULAWAN	Cultivar2	Philippines	12.8797	121.774017	7.267205049
IRGC117623	BR24	Cultivar3	Bangladesh	23.685	90.356331	7.608043765
IRGC117684	CHIEM CHANH	Cultivar4	Vietnam	14.0583	108.277199	5.997872702
IRGC117691	CO 18	Cultivar5	India	20.5937	78.96288	5.483237827
IRGC117757	IR 36	Cultivar6	Philippines	12.8797	121.774017	7.681975628
IRGC117268	IR 64-21	Cultivar7	Philippines	na	na	7.866325382
IRGC122112	IR 74371-54-1-1::C1	Cultivar8	Philippines	na	na	7.850492809
IRGC120987	IR 77298-14-1-2::IR GC117374-1	Cultivar9	Philippines	na	na	7.952878799
IRGC117758	IR 8	Cultivar10	Philippines	12.8797	121.774017	7.176140505
IRGC117829	ORYZICA LLANOS	Cultivar11	Colombia	4.57087	-74.297333	7.163793806
IRGC117841	PAPPAKU	Cultivar12	Taiwan	23.6978	120.960515	5.406225836
IRGC117880	SERATOES HARI	Cultivar13	Indonesia	-0.78928	113.921327	5.768350608
IRGC117915	TKM 6	Cultivar14	India	20.5937	78.96288	5.761919856
IRGC122272	UPLRI-7	Cultivar15	Philippines	na	na	8.600055277
