# Supplementary material for: Plant host and drought shape the root associated fungal microbiota in rice
Source: PeerJ. 2019 Sep 11;7:e7463. doi: 10.7717/peerj.7463 (PMC6744933; doi:10.7717/peerj.7463)
Supplement: Table S4 [file peerj-07-7463-s010.pdf]

Table S4. List of *Arthrinium phaeospermum* strains used in the pot experiment.

**Strain Nº**	**CBS Nº**	**Taxon name**	**Country**	**Origin**
1	CBS 142.55	*Arthrinium phaeospermum*	Japan	soil
2	CBS 463.83	*Arthrinium phaeospermum*[1]	Netherlands	*Phragmites australis*, dead culms
3	CBS 114314	*Arthrinium phaeospermum*	Iran	*Hordeum vulgare*
4	CBS 114315	*Arthrinium phaeospermum*	Iran	*Hordeum vulgare*
5	CBS 114317	*Arthrinium phaeospermum*	Iran	*Hordeum vulgare*
6	CBS 114318	*Arthrinium phaeospermum*	Iran	*Hordeum vulgare*
7	CBS 115473	*Arthrinium phaeospermum*	Hong Kong	wood
8	CBS 134073	*Arthrinium phaeospermum*	USA	*Miscanthus giganteus*

[1] Formerly known as *Arthrinium saccharicola*
